# Can pain under anesthesia be measured? Pain-related brain function using functional near-infrared spectroscopy during knee surgery

**DOI:** 10.1117/1.NPh.10.2.025014

**Published:** 2023-06-09

**Authors:** Keerthana Deepti Karunakaran, Ke Peng, Stephen Green, Christine B. Sieberg, Arielle Mizrahi-Arnaud, Andrea Gomez-Morad, David Zurakowski, Lyle Micheli, Barry Kussman, David Borsook

**Affiliations:** a Boston Children’s Hospital, Harvard Medical School, Center for Pain and the Brain, Department of Anesthesiology, Critical Care, and Pain Medicine, Boston, Massachusetts, United States; b Massachusetts General Hospital, Harvard Medical School, Department of Psychiatry, Boston, Massachusetts, United States; c Harvard Medical School, Department of Psychiatry, Boston, Massachusetts, United States; d l’Université de Montréal, Centre de Recherche du CHUM, Département en Neuroscience, Montreal, Quebec, Canada; e Boston Children’s Hospital, Biobehavioral Pain Innovation Laboratory, Department of Psychiatry and Behavioral Sciences, Boston, Massachusetts, United States; f Boston Children’s Hospital, Harvard Medical School, Division of Biostatistics, Department of Anesthesiology, Critical Care, and Pain Medicine, Boston, Massachusetts, United States; g Boston Children’s Hospital, Harvard Medical School, Department of Sports Medicine, Boston, Massachusetts, United States; h Boston Children’s Hospital, Harvard Medical School, Division of Cardiac Anesthesia, Department of Anesthesiology, Critical Care, and Pain Medicine, Boston, Massachusetts, United States; i Massachusetts General Hospital, Harvard Medical School, Department of Radiology, Boston, Massachusetts, United States

**Keywords:** anesthesia, pain monitoring, functional near-infrared spectroscopy, prefrontal cortex, surgery, beta series correlation

## Abstract

**Significance:**

Quantitative measurement of perisurgical brain function may provide insights into the processes contributing to acute and chronic postsurgical pain.

**Aim:**

We evaluate the hemodynamic changes in the prefrontal cortex (medial frontopolar cortex/mFPC and lateral prefrontal cortex) and the primary somatosensory cortex/S1 using functional near-infrared spectroscopy (fNIRS) in 18 patients (18.2±3.3 years, 11 females) undergoing knee arthroscopy.

**Approach:**

We examined the (a) hemodynamic response to surgery and (b) the relationship between surgery-modulated cortical connectivity (using beta-series correlation) and acute postoperative pain levels using Pearson’s r correlation with 10,000 permutations.

**Results:**

We show a functional dissociation between mFPC and S1 in response to surgery, where mFPC deactivates, and S1 activates following a procedure. Furthermore, the connectivity between (a) left mFPC and right S1 (original r=−0.683, $p_{\text{permutation}}=0.001$), (b) right mFPC and right S1 (original r=−0.633, $p_{\text{permutation}}=0.002$), and (c) left mFPC and right S1 (original r=−0.695, $p_{\text{permutation}}=0.0002$) during surgery were negatively associated with acute postoperative pain levels.

**Conclusions:**

Our findings suggest that greater functional dissociation between mFPC and S1 is likely the result of inadequately controlled nociceptive barrage during surgery leading to more significant postoperative pain. It also supports the utility of fNIRS during the perioperative state for pain monitoring and patient risk assessment for chronic pain.

## 1 Introduction

Surgery by its very nature produces tissue damage by activation of nociceptor nerve fibers or secondary effects via activation of inflammatory systems where specific molecules can act directly on nociceptors to induce an ongoing afferent barrage. Such ongoing nociceptive afferent signals have been shown to reach the brain under general anesthesia^1^^,^^2^ producing central sensitization of pain pathways. Having an objective measure of nociception/pain during surgery could provide the opportunity to limit the nociceptive barrages to the brain by immediate analgesic intervention and limiting analgesic use postoperatively.^1^^,^^3^^,^^4^ Importantly, it may diminish the likelihood of chronic postsurgical pain (CPSP) reported in around 30% of patients undergoing surgery.^5^ However, we are unaware of studies evaluating pain markers, specifically brain-based markers, during the perioperative state—primarily due to a lack of reliable brain imaging system suitable for a surgical setting.

We have previously reported the use of functional near-infrared spectroscopy (fNIRS) to measure evoked and ongoing nociception under general inhalational anesthesia;^1^ where we focused on two cortices: the anterior prefrontal cortex and the primary somatosensory cortex (S1).^1^^,^^4^^,^^6^^–^^8^ The anterior prefrontal cortex—divided into subregions of the medial frontopolar cortex/mFPC, dorsal lateral prefrontal cortex, and ventral lateral prefrontal cortex (together broadly labeled as lateral prefrontal cortex/lPFC)—is involved in assimilating sensory and emotional processes.^9^^,^^10^ The S1 is a brain region classically associated with nociceptive processing.^11^ Although there is no single signal in the brain that is specific to pain, the brain as a network is context-specific and could help detect the brain state relating to pain. Past studies from our group have shown a consistent functional dissociation—inverse correlation in fNIRS hemodynamic response—between S1 and mPFC/Brodmann area 10 during nociceptive stimuli, but not innocuous stimuli, in both conscious and unconscious patients.^1^^,^^6^ This functional dissociation (negative relationship between the two regions) was found during pain but not auditory stimuli and diminished following morphine and remifentanil.^4^^,^^12^ Therefore, evaluating the functional relationship between these two cortices during surgery using functional connectivity (FC) metric could identify nociceptive signaling to the brain.

Furthermore, a body of evidence indicates that several perisurgical factors may contribute to increased pain and analgesic requirements in the early postsurgical time and, importantly, to the condition of CPSP.^13^^,^^14^ For example, physical (i.e., pain) and psychological state (pain catastrophizing^15^^,^^16^ and fear of pain^17^^,^^18^) before surgery may further disrupt homeostasis and contribute to allostatic load^19^^–^^21^ following surgery, becoming significant risk factors of postoperative pain.^22^ Acute postsurgical factors, such as levels of hyperalgesia^23^ or analgesic use, have also shown to contribute to or increase the likelihood of long-term pain. Yet it is unclear how these factors can be accounted into routine clinical practice to limit problematic perioperative processes that increase the risk of ongoing acute pain and CPSP. Evaluating the brain states along with the psychological state during the pre-, intra- and postoperative stages may be the first step to understand how central nervous system changes contribute toward acute postoperative pain.

To this end, we performed continuous fNIRS scanning during the pre-, intra-, and postoperative states in 33 patients scheduled to undergo knee arthroscopy for anterior cruciate ligament repair. The psychological outlook of the patient toward pain was evaluated prior to surgery using fear of pain and pain catastrophizing questionnaires. We examined the (a) cerebral hemodynamic changes in response to surgical procedures and (b) surgery-modulated cortical FC using beta-series correlation after accounting for their preoperative psychological state. We then computed the relationship between cortical FC and acute postoperative pain levels in these patients. We hypothesized that the cortical activity in pain-related brain regions is reflective of the afferent nociceptive barrage that was not mitigated or eliminated by analgesia/regional anesthesia during surgery; and greater nociceptive barrage during surgery will contribute to greater levels of acute postoperative pain. Additionally, we examined baseline brain changes before and after surgery to understand how brain state postsurgery relates to postoperative pain levels and presurgical outlook toward pain.

## 2 Materials and Methods

### 2.1 Participants

Data were collected from 33 participants undergoing arthroscopic knee surgery under general anesthesia. Eligible individuals were between 12 and 25 years of age. Individuals were excluded if they had structural brain disorders, significant medical disorders, such as diabetes, cardiac disease, autism, developmental delay, or cancer, or a history of smoking. Patients from whom reliable NIRS measures could not be obtained during an initial signal test, who could not keep their head still for 200 consecutive seconds, or who could not cooperate or understand the nature of the study were also excluded. Informed consent was obtained from the participants or their parent/guardians prior to the study, and assent was obtained from minor children. All study procedures were approved by the Institutional Review Board of Boston Children’s Hospital, Harvard Medical School, Boston, United States.

### 2.2 Psychological and Pain Measures

Preoperative and postoperative pain intensity was assessed on an 11-point numeric rating scale, where 0 = no pain and 10 = worst possible pain.^24^ The numeric rating scale is a valid measure of pain severity in children and adolescents.^25^^,^^26^ The pain catastrophizing scale-child version is a 13-item self-report measure that assesses pain-related negative thinking and includes the subscales of magnification, rumination, and helplessness.^27^^,^^28^ For this questionnaire, the participants rated the items on a 5-point scale yielding a maximum score of 52, with higher scores indicating greater distress. Fear of pain questionnaire-child version comprises of 24 items and assesses pain-related fear and avoidance behaviors.^29^ Items were rated on a 5-point scale from 0 (strongly disagree) to 4 (strongly agree), where higher scores indicate higher levels of fear and anxiety toward pain. These measures have strong internal consistency and construct validity, which supported by the questionnaire is strongly associated with child somatization, anxiety, catastrophizing, and functional disability.

### 2.3 FNIRS Acquisition

**Fig. 1:**
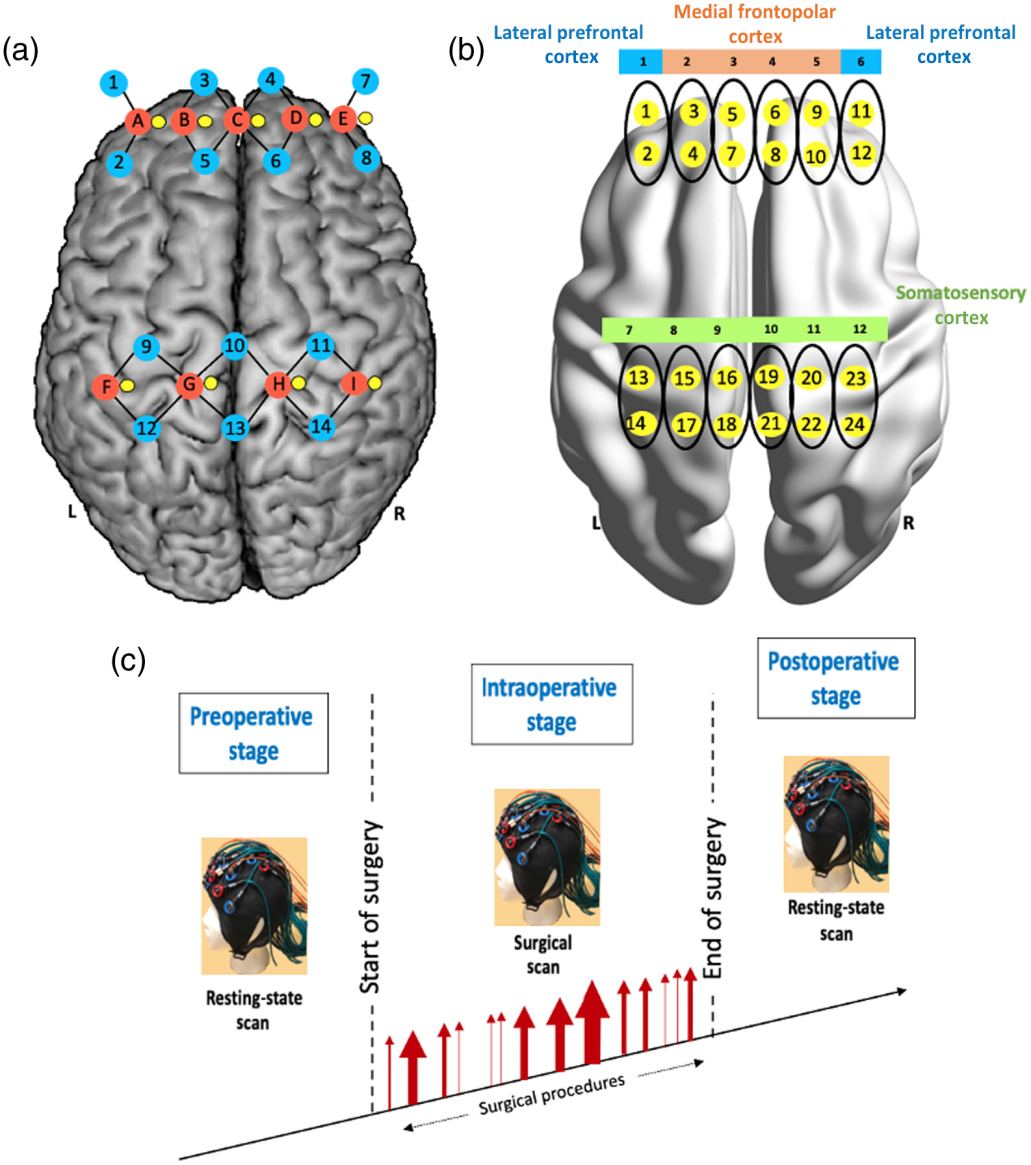
FNIRS optode and channel layout. (a) Brain map of customized optode placement where red represent optical sources, blue represent long-separation detectors (3 cm from the source), and yellow represent the short-separation detectors (0.8 cm from the source). A detector–source pair forms a channel and is represented by the black lines connecting the sources and detectors. The short separation channels measuring extracerebral hemodynamic changes are not shown. (b) Brain map of channel locations separated into 12 regions of interest by averaging two channels (highlighted by black) color coded based on the cortical regions of interest, mFPC, lPFC, and somatosensory cortex. (c) Experimental paradigm for fNIRS acquisition. Each visit consisted of preoperative, intraoperative, and postoperative stages. The time stamps of the surgical procedures during surgery were noted.

The fNIRS recording was sampled at a frequency of 25 Hz using a continuous wave fNIRS system (CW6, NIRSOptix by TechEn, Massachusetts, United States) at 690 and 830 nm wavelengths. A custom-designed head probe consisting of 9 sources, 14 long-separation detectors (placed at a distance of 3 cm), and 9 short-separation detectors (placed at a distance of 0.8 cm) were used to record signals from the bilateral prefrontal and somatosensory cortices [Fig. 1(a)]. A channel, denoted by the black lines in Fig. 1(a), is the light intensity recording from a source–detector pair. Out of the 33 channels, 24 channels recorded the hemodynamic changes from the cortex [shown in Figs. 1(a) and 1(b)], while the remaining 9 channels recorded hemodynamic changes from extracerebral tissue [not shown in Fig. 1(b)]. The channels were further grouped into 12 regions of interest, where the average of two channels in the anterior–posterior direction [highlighted in Fig. 1(b)] was defined as a region of interest (referred to as regions in this study). The 12 regions based on their anatomical location were broadly categorized into lPFC, mFPC, and S1, denoted by the colors in Fig. 1(b).

### 2.4 FNIRS Protocol

The first fNIRS recording was performed while the patient was being prepared for surgery in the preoperative room. Once the clinicians completed their routine preoperative procedures, head probes were placed on the patient’s scalp, and optode-scalp contact was confirmed by a heart rate signal in every channel. Following the installation, a 10-min resting-state scan was collected. The patients were instructed to stay awake, silent, and remain as still as possible. The anatomical location of the optodes was maintained between recordings by transferring the patient to the operating room while still wearing the head probe secured to the fNIRS system.

In the operating room, the quality of the data was once again evaluated prior to anesthetic induction by establishing the heart rate signal in the different cortices. The patients were then induced using a standard anesthetic regimen, where patients were premedicated with intravenous 2 mg midazolam and induced using propofol (median 200 mg [min 150 mg to max 300 mg]) along with fentanyl or sufentanil. The detailed anesthetic protocol of this patient cohort is discussed in our prior papers.^30^^,^^31^ In addition to general anesthesia, peripheral nerve block with ropivacaine was administered to the adductor canal on the ipsilateral limb of 11 patients using the guidance of an ultrasound. A lateral femoral cutaneous nerve block was added to the adductor canal block in one patient. After induction, continuous fNIRS data were collected from the patient for the duration of the surgery (minimum of 25 min to maximum of 5 h). Procedures during surgery (e.g., incisions, manipulations, or injections) were timestamped in the data. Painful surgical events were defined as procedures that manipulated the bone, tissue, or skin. The most common surgical events included injection, incision, shaving, scraping, poking, drilling, hammering, cutting, burning, and sutures. Following surgery, the patient was moved to the postoperative recovery room while still connected to the fNIRS system. A resting-state scan was acquired for a duration of 5 min during emergence from anesthesia.

### 2.5 FNIRS Preprocessing

All data and statistical analyses were performed on the MATLAB R2019b platform using in-house scripts. First, the data were visually inspected to ensure acceptable signal quality. Acceptable signal quality was defined as a (1) minimum raw signal intensity of $10^{3}$ and (2) visible cardiac fluctuations in the time series or a frequency component corresponding to heart rate in the power spectrum of the raw time series (∼1 to 2 Hz). A subject was considered to have poor data quality if a cardiac component was not present in the raw timeseries of at least four channels in each medial prefrontal cortex and somatosensory cortex. For subjects with acceptable data quality, the raw data from the 33 channels of each participant were converted from intensity measures to optical density measures. Correction for head-motion artifacts was then performed using a wavelet-based algorithm.^32^ Next, physiological noise sources, such as heart rate and respiration were removed using a third-order bandpass filter at a frequency range of 0.01 to 0.3 Hz for intraoperative data and 0.01 to 0.1 Hz for preoperative and postoperative resting-state data. The filtered optical density measures of each run were converted to oxyhemoglobin, deoxyhemoglobin, and total hemoglobin concentration changes using the Beer–Lambert law via the *hmrOD2conc* function from the Homer2 toolbox.^33^ The hemoglobin concentration time series for each channel was regressed using a linear temporal regression of the first three principal components derived from the nine short-separation channels (physiological signals). This was performed for each oxyhemoglobin, deoxyhemoglobin, and total hemoglobin time series in each run to remove the effect of extracerebral tissue on cortical activity. The residuals of the temporal regression—for each oxyhemoglobin, deoxyhemoglobin, and total hemoglobin time series—were corrected for linear and nonlinear drifts using a third-order polynomial fit. Due to greater sensitivity to cerebral hemodynamic changes,^34^ only oxyhemoglobin and total hemoglobin time series were used for further data analysis.

### 2.6 Data Analysis

#### 2.6.1 Resting-state FC

The resting-state FC during preoperative and postoperative states was computed using Pearson’s r pair-wise correlation of the 12 regions. This was performed by correlating the hemoglobin timeseries of each region with the remaining 11 regions to form a 12×12 matrix for each oxyhemoglobin and total hemoglobin measures. The Pearson’s r correlation values were converted to Fisher-z scores using Fisher r to z transformation before statistical analysis. The mean resting-state FC matrix during preoperative and postoperative states was calculated as the average of individual matrices from all patients.

#### 2.6.2 Cortical response to surgical procedures

The block-averaged hemodynamic response of a cortical region, i.e., the changes in hemoglobin concentration to surgical procedures, was computed by averaging 30 s of time series after a surgical procedure (also called as an event) across all procedures a patient underwent during the surgery. Before averaging across the events, the time series following an event was normalized using the 5 s of timeseries before an event. This was performed for every region in each patient. The group averaged hemodynamic response using oxyhemoglobin and total-hemoglobin time series were obtained by averaging the block-averaged hemodynamic response across all patients for every cortical region.

#### 2.6.3 Intraoperative functional brain connectivity

FC during surgery was computed using the Beta Series Correlation technique, where we calculate the pairwise correlation of trial-by-trial task activation measures of different brain regions.^35^^,^^36^ Of note, the task in this study is the surgical intervention. Originally applied for task-fMRI data to compute task-related FC, the beta series correlation method is performed using ordinary least squares estimate of each trial/stimulus (regressor-1) against all other trials/stimuli (regressor-2). The stimulus function for a given trial is generated by assigning 1s during the duration of a trial and 0s for everything else. Similarly, the stimulus function of the remaining trials is generated by assigning 1s for the duration of all the remaining trials and 0s for everything else. The stimulus functions are then convolved with a double-gamma hemodynamic response function to generate regressor-1 and regressor-2 and inputted into an ordinary least squares model. This is repeated for every trial to obtain a beta estimate of activation per trial. A trial was defined as a surgical procedure lasting for at least 2-s duration and occurred 30 s apart. Therefore, the size of the resulting beta series of a patient is equal to the total number of trials (i.e., surgical events) in that patient. A Pearson’s r correlation of the beta series between all 12 regions was performed to calculate the intraoperative procedure-related connectivity matrix (size of 12×12) of a patient. The Pearson’s r correlation values were converted to Fisher-z scores using Fisher r to z transformation before performing statistical analysis.

### 2.7 Statistical Analysis

The statistical analyses described below were performed separately for oxyhemoglobin and total hemoglobin time series.

(1) *Preoperative versus postoperative resting-state FC.* The preoperative and postoperative FCs were compared to identify differences associated with the intraoperative state, likely emerging from effects of anesthesia, analgesia, and surgery. Before comparing the resting-state FC measures of the unique connections (12×11/2=66 connections), the normality of the distribution of group resting-state FC measures was tested using a single sample Kolmogorov–Smirnov test. If the resting-state FC measures of a region-to-region connection was normal, a paired-sample t-test was performed comparing the Fisher-z scores of resting-state FC in the 18 patients at preoperative and postoperative states. If the resting-state FC measures of a region-to-region connection was not normal, a paired two-sided Wilcoxon signed rank test was performed to compare the Fisher-z scores of resting-state FC in the 18 patients at preoperative and postoperative states. A statistical threshold of p<0.05 with a Benjamini–Hochberg false-discovery rate approach of α=0.05 was used to reduce type-I errors from multiple comparison problems.^37^ Region-to-region connections that were statistically different between the two states were further correlated with preoperative fear of pain, pain catastrophizing measures, and postoperative pain levels.
(2) *Intraoperative FC and acute postoperative pain levels*. Relationship between intraoperative FC of each of the 66 connections and postoperative pain levels were determined using a simple Pearson’s r correlation. The significance of this relationship was determined using a randomization test with 10,000 permutations by randomly pairing the FC samples with postoperative pain levels. Effect of psychological variables (fear of pain and pain catastrophizing) on brain connectivity measures was mitigated using a partial correlation analysis, and the residuals were used for permutation tests. The p-value was calculated as the proportion of permutation tests that resulted in a Pearson’s r value greater than or equal to the original correlation between the two variables. A Benjamini–Hochberg false-discovery rate correction with an α=0.05 was performed to account for type-I errors due to multiple comparison problems.^37^

## 3 Results

### 3.1 Participants

Six of the 24 patients were excluded from the analysis due to poor data quality. The demographic information of the remaining 18 patients is provided in Table 1.

**Table 1:** Demographic and procedural data (n=18).

Patient	Age	Gender	Side of surgery	Nerve block (Y/N)	Preoperative pain level	Postoperative pain level	Fear of pain	Pain catastrophizing
10	17	F	R	Y	NA	6	50	18
12	19	F	L	N	NA	0	63	20
13	17	F	R	N	0	0	38	13
15	13	F	R	Y	0	4	39	16
16	14	M	R	Y	NA	7	24	6
17	16	F	R	N	3	6	58	24
18	18	M	R	Y	2	8	78	29
19	19	M	L	Y	0	6	39	14
22	22	F	R	Y	0	2	25	11
23	14	F	R	Y	0	2	64	17
24	23	M	L	N	5	6	47	12
25	16	M	L	Y	0	4	26	1
26	25	F	L	Y	NA	5	46	7
27	17	F	R	N	NA	3	26	12
28	22	M	R	N	0	5	53	21
30	19	F	L	Y	0	2	55	9
31	21	F	R	N	0	6	24	0
32	16	M	R	Y	0	6	49	5

Note: NA indicates that preoperative pain level for that patient is not available.

### 3.2 Self-Reported Pain

No significant relationship was found between acute postoperative pain levels and whether the patient received peripheral nerve block using ANOVA (F(1,16)=0.784, p=0.389). ANOVA was also performed to identify if there was a significant association between postoperative pain levels and sex. Male patients (n=7) reported greater postoperative pain levels than female patients (F(1,16)=8.187, p=0.011). No notable association was found between acute postoperative pain level and side of surgery (F(1,16)=0.392, p=0.540).

### 3.3 Pre- versus Postoperative Resting-State FC

**Fig. 2:**
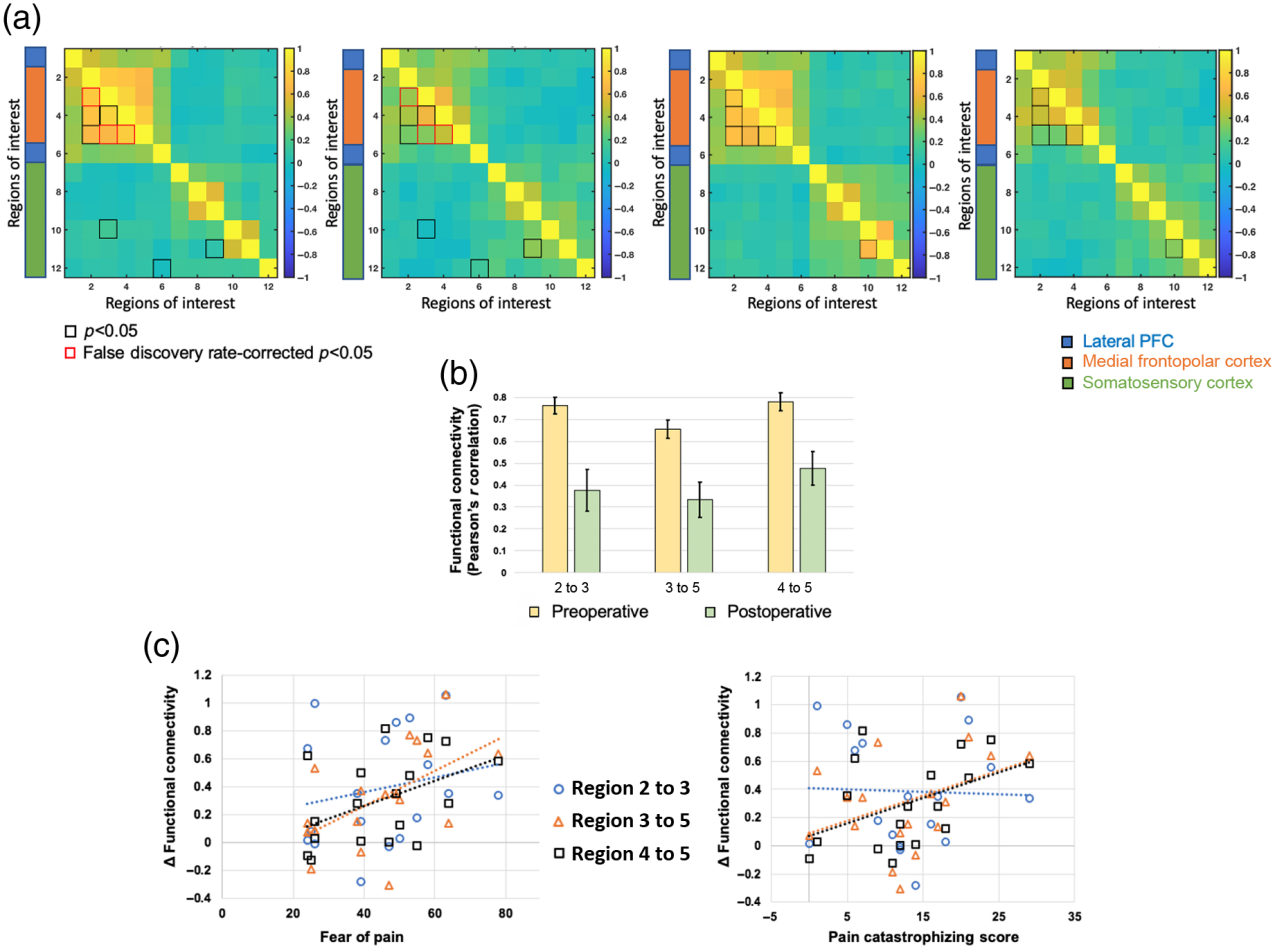
Pre- versus postoperative resting-state FC. (a) Mean resting-state FC between the 12 regions during preoperative versus postoperative: left, oxyhemoglobin and right, total hemoglobin. The black squares indicate connections from paired sample t-tests that are significant at uncorrected p<0.05. The red squares indicate connections significant at false discovery rate-corrected p<0.05. The color bar represents Pearson’s r correlation value of connectivity, where warmer colors denote a positive r value, whereas cooler colors denote a negative r value. (b) Bar plots of mean FC between left lPFC and left mFPC (regions 2 to 3), left mFPC and right mFPC (regions 3 to 5), and within right mFPC (regions 4 to 5) during the pre- and postoperative states. (c) Relationship between fear-related constructs of pain (fear of pain and pain catastrophizing) and difference in pre- versus postoperative resting-state FC. Higher pain-related fear and catastrophizing scores were associated with greater decrease in resting-state FC after surgery. Region 1, left lPFC; regions 2 and 3, left mFPC; regions 4 and 5, right mFPC; region 6, right lPFC; regions 7 to 9: left S1; and regions 10 to 12, right S1.

The duration of resting-state scans differed from patient to patient due to unforeseen medical interventions, however, a minimum of 5 min of resting-state data was acquired from all patients. Therefore, the resting-state FC matrix of oxyhemoglobin, and total hemoglobin was calculated using 4 min of data after removing the first minute of the time series to allow the signal to stabilize. The statistical tests comparing the resting-state FC using oxyhemoglobin concentration changes during pre- and postoperative states revealed a decrease in resting-state FC within the regions of mFPC during the postoperative state [highlighted in Fig. 2(a)-left panel], particularly within left mFPC (regions 2 to 3, $p=8.192\times 10^{-4}$, d=0.17), between left mFPC and right mFPC (regions 3 to 5, p=0.0014, d=0.27), and within right mFPC (regions 4 to 5, $p=6.897\times 10^{-4}$, d=0.74). The average FC of these regions during the pre- and postoperative states is shown in Fig. 2(b). All the three connections were statistically different after false discovery rate-based correction at p<0.05 (uncorrected-p threshold = 0.0014). Further correlating the difference in resting-state FC of these regions during pre- and postoperative states with fear of pain and pain catastrophizing scores prior to surgery indicated a positive association between the two for regions in right mFPC [regions 4 to 5, Pearson’s r correlation of ΔFC and fear of pain = 0.459, p=0.054; Pearson’s r correlation of ΔFC and pain catastrophizing = 0.449, p=0.061, see Figs. 2(c) and 2(d)]. No notable relationship was observed between these FC measures and postoperative pain levels (p>0.05). A similar trend in mean resting-state FC pattern was observed using total hemoglobin time series, shown in Fig. 2(a)-right panel, however, no statistical differences were noted between the preoperative and postoperative states.

### 3.4 Cortical Response to Surgery

**Fig. 3:**
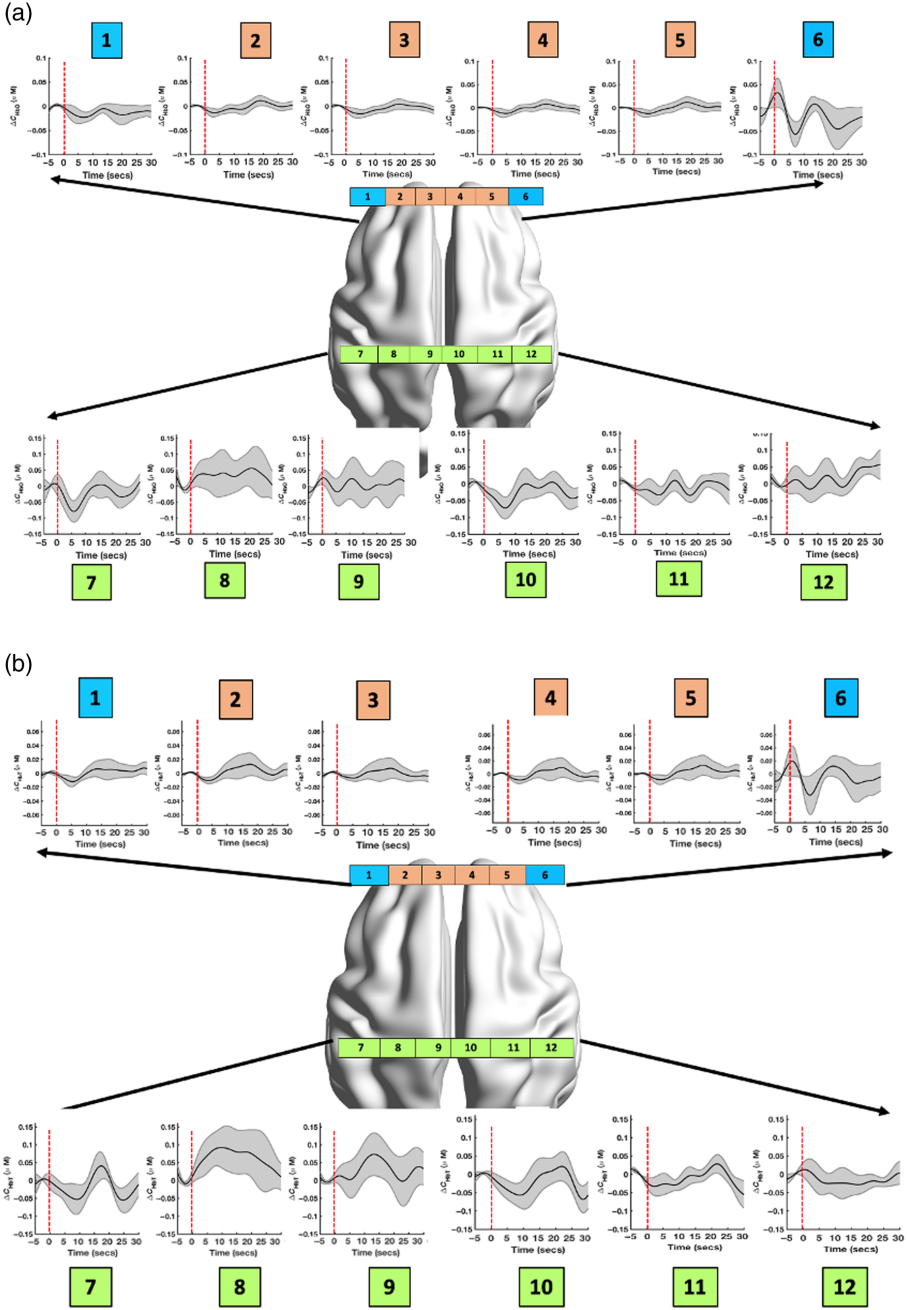
Surgical procedure-related hemodynamic response during intraoperative state. Group averaged hemodynamic response to surgical procedures in the 12 regions using (a) oxyhemoglobin time series and (b) total hemoglobin time series. The red line indicates the beginning of surgical procedure. Region 1, left lPFC; regions 2 and 3, left mFPC; regions 4 and 5, right mFPC; region 6, right lPFC; regions 7 to 9, left S1; and regions 10 to 12, right S1.

The group hemodynamic response using oxyhemoglobin concentration changes and total hemoglobin concentration changes (details of the procedure provided in Table 1) in the 18 patients is shown in Figs. 3(a) and 3(b). The group-averaged hemodynamic response using oxyhemoglobin concentration changes displays a decrease/deactivation in mFPC regions in the first 10 s after stimuli, followed by an increase at ∼20  s after stimuli. In contrary, an increase/activation was observed in S1 regions following the start of stimuli. The group-averaged hemodynamic response using total hemoglobin concentration changes—sum of oxyhemoglobin and deoxyhemoglobin concentration changes—indicates a net increase (resembling a typical hemodynamic response) to stimuli in all regions, although the variance in group activation (i.e., the standard error of mean at 0 to 20 s) of mFPC region is larger than the response using oxyhemoglobin concentration changes.

### 3.5 Intraoperative Cortical Functional Connectivity and Acute Postoperative Pain

**Fig. 4:**
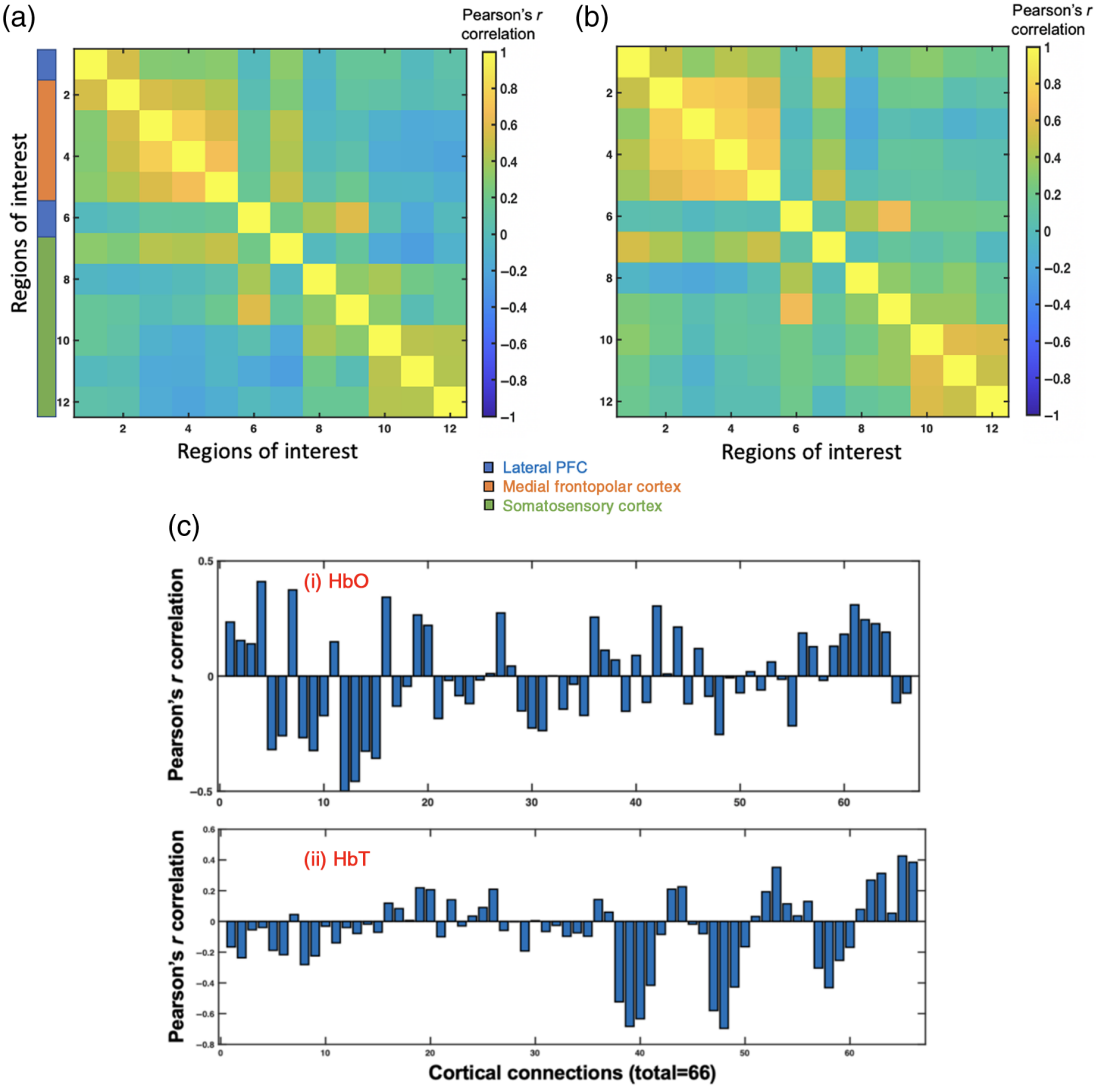
Intraoperative surgical procedure-related FC using beta-series correlation: Group-level FC modulated by surgical procedures calculated using (a) oxyhemoglobin and (b) total hemoglobin time series. Pairwise correlation of beta estimates of activation to surgical procedures between regions were inferred as intraoperative procedure-related FC. Color bar represents Pearson’s r correlation measure where hotter colors are positive correlation, and cooler colors indicate negative correlation. (c) Relationship between cortical FC during surgery and postoperative pain levels in 66 cortical connections shown above, computed using (i) oxyhemoglobin concentration data and (ii) total hemoglobin concentrations. Region 1, left lPFC; regions 2 and 3, left mFPC; regions 4 and 5, right mFPC; region 6, right lPFC; regions 7 to 9, left S1; and regions 10 to 12, right S1.

**Fig. 5:**
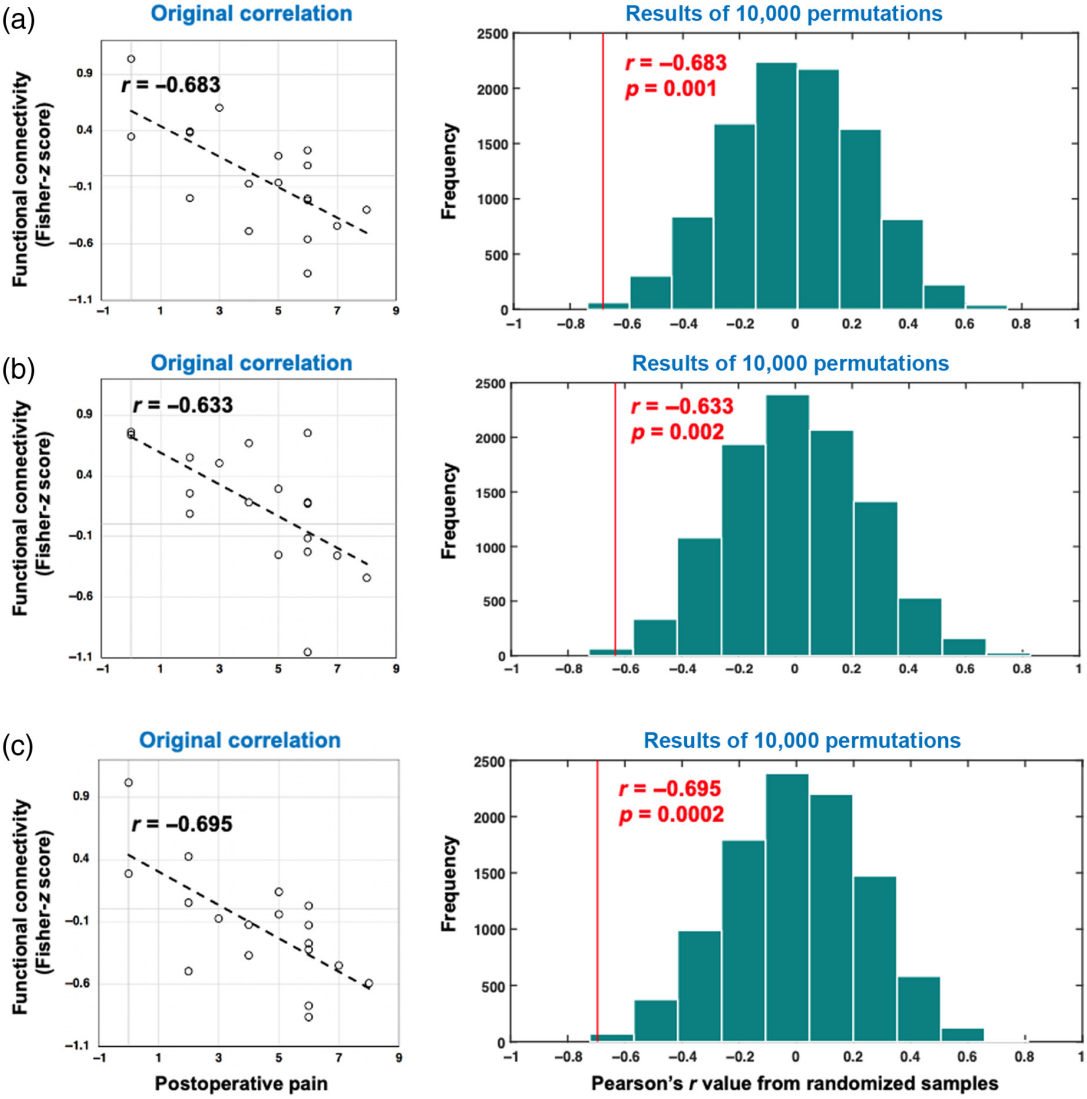
Intraoperative surgical procedure-related FC and postoperative pain levels: 10,000 permutation tests computing the correlation between procedure-related FC using total hemoglobin concentration changes and postoperative pain levels resulted in three functional connections that were statistically significant at false discovery rate-p<0.05 (uncorrected-p=0.0017), (a) left mFPC and right medial S1 (regions 3 to 10), (b) left mFPC and right medial S1 (regions 3 to 10), and (c) left mFPC and right medial S1 (regions 3 to 10). Left panels show the original scatter plot between FC and postoperative pain levels. Right panels show the distribution of Pearson’s r values of the 10,000 permutation tests where the red line represents the original correlation value and red text shows the original Pearson’s r value and p-value from permutation tests.

Output of beta series correlation represent the FC between cortices modulated by the surgical procedures. The mean intraoperative FC of 18 patients using oxyhemoglobin and total hemoglobin time series is presented in Figs. 4(a) and 4(b). The intraoperative FC using both oxyhemoglobin and total hemoglobin exhibited dense positive connections within both prefrontal cortex and somatosensory cortex in response to surgical procedures, i.e., the response within these individual cortices were highly homogenous. Using a pairwise correlation analysis of all 66 functional connections, we found that the intraoperative FC of oxyhemoglobin concentration changes within the mFPC/Brodmann area 10 was negatively associated with postoperative pain levels [see Fig. 4(c)(i)]; whereas the intraoperative FC of total hemoglobin concentration changes between mFPC and S1 was negatively associated with postoperative pain levels [see Fig. 4(c)(ii)]. The significance of this relationship was examined using a nonparametric test with 10,000 permutations. Randomization of correlation between pain levels and FC using oxyhemoglobin concentration changes revealed no statistically significant association between the two after multiple comparison correction. Randomization of correlation between pain levels and FC using total hemoglobin concentration changes revealed a negative association between FC measures of the cortex and postoperative pain levels at false discovery rate-corrected p<0.05 (uncorrected-p threshold = 0.0017). The functional connections that were negatively correlated with postoperative pain levels were (a) left mFPC and right S1 [regions 3 to 10, original Pearson’s r=−0.683, $p_{\text{permutation}}=0.001$, Fig. 5(a)], (b) right mFPC and right S1 [regions 4 to 10, original Pearson’s r=−0.633, $p_{\text{permutation}}=0.002$, Fig. 5(b)], and (c) left mFPC and right S1 [regions 3 to 11, original Pearson’s r=−0.695, $p_{\text{permutation}}=0.0002$, Fig. 5(c)]. $p_{\text{permutation}}$ value was computed as the proportion of 10,000 iterations that yielded a correlation value smaller or equal to the original correlation value. As observed in Fig. 5, the negative procedure-related FC between the two cortices (i.e., mFPC and S1) during surgery was associated with higher reported pain levels during postoperative state (VAS pain levels of 5 or more).

## 4 Discussion

Using fNIRS, we report cortical response and FC of S1 and prefrontal cortices during the following states: (1) *pre- versus postoperative state*: the patient’s brain state prior to surgery versus acute postoperative state reflecting the surgery and anesthesia and (2) *intraoperative state*: brain state modified by surgically induced nociceptive drive occurring during surgery. The approach allowed us to determine cortical changes during surgery that relate to acute postoperative pain levels, where patients with greater functional dissociation between mPFC-S1 in response to procedure had greater postoperative pain. We believe this could be due suboptimal or inadequate analgesic coverage during the procedure that could be overcome using an objective pain monitoring system such as fNIRS.

### 4.1 Pre- versus Postoperative State-Potential Effects of Anesthesia

The resting-state FC before surgery was compared with the postoperative state. The FC of mFPC between and within the left and right hemispheres was significantly reduced during the postoperative state when compared to the preoperative awake state. Imaging studies have evidenced anterior prefrontal cortex to uniquely contribute to executive functioning depending on the subregion. For example, the mFPC is thought to play a key role in the default mode network (DMN) during unconscious processing, and internal modes of cognition, such as self-oriented thoughts/tasks and autobiographic memory retrieval.^38^ At rest, the mFPC region is positively connected to regions of posterior cingulate cortex, precuneus, inferior parietal cortex, hippocampus, etc. A decrease in the FC of channels within the mFPC is suggestive of a decrease in the local mFPC activity and may be attributed to the anesthetic drug and reduced level of consciousness while emerging from anesthesia. One study using integrated local correlation, a measure of neural synchrony in neighboring neural anatomical regions, showed a decrease in prefrontal cortex during light sedation under sevoflurane.^39^ fMRI studies using midazolam^40^ and propofol^41^ to induce light sedation reported no changes in the prefrontal cortex connectivity of the DMN. Surgical trauma and analgesia may also affect the postoperative brain state, although it is not possible to isolate the cause. Additionally, the observed difference in FC between the preoperative and postoperative states was positively associated (but not statistically significant) to fear of pain and pain catastrophizing levels prior to surgery, where higher levels of fear-related emotions toward pain were associated with a greater decrease in FC of mFPC during wakefulness. Both catastrophizing and fear/anticipation of pain have shown to heighten pain perception and emotional processing of painful stimuli.^42^^,^^43^ It is unclear if premorbid risk factors, such as psychological state, could affect the acute postoperative brain state in a way that predisposes them to central sensitization following surgery. Further work is needed to understand how psychological and surgical factors may be used to predict the incidence of severe acute and chronic postoperative pain.

### 4.2 Intraoperative State

Noxious surgical procedures under anesthesia were associated with deactivation of mFPC (immediate decrease in oxyhemoglobin concentration changes following stimuli) and activation of S1 (sustained increase in oxyhemoglobin concentration changes following stimuli). A similar response was also observed using the total hemoglobin measure (Fig. 3). The correlation in beta estimates of these regions’ response to surgical procedures was inferred as cortical integration modulated by surgery. Examining the relationship between FC (using total hemoglobin) of the 12 regions and postoperative pain levels using permutation tests revealed that connectivity between bilateral mFPC and right S1 during surgery was negatively associated with postoperative pain levels. Specifically, negative connectivity between mFPC and S1 during surgical procedures was associated with higher postoperative pain levels (VAS>5). As previously reported, nociception but not innocuous sensory stimuli was found to result in negative activation in mFPC and positive activation in S1 in individuals irrespective of their state of consciousness (awake, sedated, or anesthetized).^1^^,^^4^^,^^7^^,^^8^ Hence, an increased negative correlation between mFPC and S1 activity during surgery may be reflective of greater nociceptive barrage to the cortex resulting from (a) significant tissue damage and nerve injury, (b) incomplete regional anesthesia, (c) inadequate analgesia, or (d) some combination of the above. The clinical significance of these findings is twofold. Surgery modulated cortical connectivity measures could be used as a “nociceptive signature” during surgery to inform the anesthesiologists. However, the next step is to validate this approach in a larger clinical sample and understand the specificity and sensitivity of the marker. If successful, neural networks and machine learning algorithms could be developed to identify these markers (surgery-modulated connectivity) in real time. Second, invasive procedures, such as orthopedic surgeries that involve increased trauma to nerves, among other factors, are a significant risk factor for high acute postoperative pain and the development of CPSP.^13^^,^^44^ Surgery-related cortical activity can be useful in estimating the net surgical load that contributes toward the development of CPSP in a patient, to inform the surgeon of high-risk individuals. However, since the long-term pain status of the current cohort is unavailable, it is unclear whether the acute postoperative pain level correlates with the long-term pain status of the cohort.

### 4.3 Implications for Surgically Induced Pain and its Prevention

An estimated one in five individuals undergoing surgery is found to develop CPSP with significant implications on the quality of life and health care costs.^45^ Both surgical and nonsurgical risk factors of CPSP have been well defined.^13^^,^^46^^,^^47^ Having surgery itself poses as a risk factor for neuropathic pain. The preoperative brain state as it relates to pain levels (pain sensitivity), psychological state (fear of pain, catastrophizing, anxiety, and depression), and other biological factors (age and biological sex) may confer risk factors for the development of CPSP. The ability to evaluate these factors as potentiators of intraoperative effects of nociceptive drive on central sensitization could enable the identification of at-risk individuals. This provides the opportunity to employ minimally invasive procedures and deliver pre-emptive analgesia and regional anesthesia to individuals with increased susceptibility to peripheral and central sensitization. The nature of surgery and the extent of surgical trauma are also related to acute postoperative pain; individuals with high acute postoperative pain have a two to threefold increase in risk of developing CPSP.^48^ However, current anesthetic standards of care do not objectively evaluate the intraoperative cortical activity induced by surgical trauma affecting nerves directly and indirectly through inflammatory processes. Cortical-based measures of the intraoperative state may provide biomarkers for (1) the anesthesiologist to deliver appropriate intervention during surgery such as multimodal analgesia to reduce inflammatory processes and (2) the surgeon regarding postsurgical outcomes, such as postoperative pain levels that are potential indicators of pain chronification. In the event of chronification, the technique could also be used to understand the cortical mechanisms relating to CPSP, and its resolution following treatment to understand drug mechanisms and treatment efficacy.^9^

### 4.4 fNIRS as an Intraoperative Monitoring System

fNIRS technique, due to its noninvasive nature, ease of setup, portability, and flexibility is an excellent neuroinvestigative tool for surgeons and anesthesiologists. Concurrent physiological, autonomic, and cerebral hemodynamic monitoring (providing greater specificity of pain measures) is already made possible by newer systems in the market, such as NIRx Sport, NIRSIT, and NINJANIRS. These newer systems offer compact hardware, and superior scalp positioning, that can be easily implemented into routine surgical practice. Optodes may be placed on the forehead and scalp of patients without discomfort, or interference from head movement or other electrical medical equipment, similar to how a bispectral index is used for depth of anesthesia. Real-time data processing software, such as Turbo-satari by NIRSx or NINJANIRS provides platforms for neuroscientists/bioengineers to develop fNIRS-based applications, such as neurofeedback, brain computer interface, and bedside patient monitoring systems for various patient populations.

### 4.5 Caveats

This study is limited by a number of caveats: (1) *Numbers of patients.* We recruited a total of 33 patients, however, due to the variability in optode location, and poor quality, or incomplete data, only 18 out of the 33 patients were used. (2) *Nature of surgical and anesthesia paradigm*. The anesthetic/analgesic regimen, the duration, nature, and complexity of surgery were unique to a patient. Accounting for these secondary factors is beyond the scope of this study. Future efforts from our group will include understanding the effect of regional anesthesia and the development of an analgesic index. (3) *Secondary factors contributing to the perioperative state*. The preoperative state may be confounded by procedures surrounding the patient, such as interactions with clinical staff. Intraoperative and postoperative states are bound to be confounded by the differing levels of surgical procedures between patients. However, these factors are often impossible to control during surgery. (4) *Effects of biological sex.* Due to the collinearity between sex and pain levels, where male patients report higher postoperative pain levels than female patients, the sex of the patient was not included as a regressor. One of our immediate future steps is to understand the effects of sex on perioperative brain state as female patients in general have been shown to have greater pain sensitivity and are at increased risk of severe pain.^49^ (5) *Physiological and autonomic factors.* We do not evaluate for changes in the physiological and autonomic variables, such as blood pressure, heart rate, or respiration, during the intraoperative period. However, understanding the relationship between these factors with the fNIRS measures would be essential to fully model nociception and analgesia in the brain. (6) *Other risk factors of CPSP.* As such, the current study does not account for other risk factors such as genetic mutations, psychological vulnerability, and other comorbidities. (7) *Technical issues*. fNIRS due to its inherent limitations only allow recording of the superficial cortex with limited spatial resolution and noise introduced due to scalp hair, and movements during surgery. Multimodal systems with both fNIRS and electroencephalography (EEG) may be better suited for intraoperative monitoring as EEG allows measurement of depth of anesthesia and access to deeper brain regions.^50^ It is also unclear how the ambient light in the operating room may influence the intensity of the fNIRS signal, although for this study, the lights were targeted away from the head. Still measures to reduce the contamination of ambient light are recommended. (8) *Methodological considerations*. It is worth noting that no additional preprocessing, such as prewhitening or precoloring, was performed to account for structured or colored noise in the fNIRS data. fNIRS, due to its high sampling rate and susceptibility to physiological noise, may require additional steps to account for colored noise while performing ordinary least square estimates.^51^ However, as beta series correlation evaluates trial-to-trial variability by correlating the estimates across all trials between regions, we believe the error noise, even if colored, will not significantly affect the relationship between channels i.e., assuming these are global/systemic noise. Added PCA-based regression of short-separation channel signals would have mitigated systemic physiological noise sources. Nonetheless, we acknowledge the need to understand how these different methods affect the validity of the BSC approach.

## 5 Conclusions

To-date, ongoing monitoring of brain systems during the perisurgical period has been done using EEG-based systems to evaluate depth of anesthesia.^52^^–^^54^ There are no studies that evaluate pain/nociception across the perisurgical period that can provide insights into how brain states may predict a patient’s responsivity to surgically induced processes that may produce central sensitization and consequently enhanced postoperative pain levels and potentially even CPSP.^13^^,^^55^ Here we provide further support for measures of mFPC and S1 changes that correlate with responses in awake individuals, suggesting that even under general inhalational anesthesia similar pain pathways are activated by surgical induced nociceptive drive. Specifically, the FC of these regions in response to surgery may give anesthesiologists an objective measure of pain during the intraoperative period and even help predict postoperative pain. The development of fNIRS systems that are adaptive in the surgical environment and allow for real-time evaluation of nociceptive events may contribute to amelioration of intraoperative pain through the provision of adequate levels of analgesics.

## Code, Data and Materials Availability

Please email Keerthana Deepti Karunakaran (keerthanadeepti@gmail.com) or David Borsook (dblabore@gmail.com) to request access to this dataset or codes.
